# Test of cure, retesting and extragenital testing practices for *Chlamydia trachomatis* and *Neisseria gonorrhoeae* among general practitioners in different socioeconomic status areas: A retrospective cohort study, 2011-2016

**DOI:** 10.1371/journal.pone.0194351

**Published:** 2018-03-14

**Authors:** Juliën N. A. P. Wijers, Geneviève A. F. S. van Liere, Christian J. P. A. Hoebe, Jochen W. L. Cals, Petra F. G. Wolffs, Nicole H. T. M. Dukers-Muijrers

**Affiliations:** 1 Department of Medical Microbiology, Maastricht University Medical Center, Care and Public Health Research Institute (CAPHRI), Maastricht, the Netherlands; 2 Department of Sexual Health, Infectious Diseases and Environmental Health, Public Health Service South Limburg, Heerlen, the Netherlands; 3 Department of Family Medicine, Care and Public Health Research Institute (CAPHRI), Maastricht University, Maastricht, the Netherlands; University of California, San Francisco, Universit of California, Berkeley and the Childrens Hospital Oakland Research Institute, UNITED STATES

## Abstract

**Background:**

For *Chlamydia trachomatis* (CT), a test of cure (TOC) within 3–5 weeks is not recommended. International guidelines differ in advising a *Neisseria gonorrhoeae* (NG) TOC. Retesting CT and NG positives within 3–12 months is recommended in international guidelines. We assessed TOC and retesting practices including extragenital testing in general practitioner (GP) practices located in different socioeconomic status (SES) areas to inform and optimize local test practices.

**Methods:**

Laboratory data of 48 Dutch GP practices between January 2011 and July 2016 were used. Based on a patient’s first positive CT or NG test, the proportion of TOC (<3 months) and retests (3–12 months) were calculated. Patient- and GP-related factors were assessed using multivariate logistic regression analyses.

**Results:**

For CT (n = 622), 20% had a TOC and 24% had a retest at the GP practice. GP practices in low SES areas were more likely to perform a CT TOC (OR:1.8;95%CI:1.1–3.1). Younger patients (<25 years) were more likely to have a CT TOC (OR:1.6;95%CI:1.0–2.4). For CT (n = 622), 2.4% had a TOC and 6.1% had a retest at another STI care provider. For NG (n = 73), 25% had a TOC and 15% had a retest at the GP practice. For NG (n = 73), 2.7% had a TOC and 12.3% had a retest at another STI care provider. In only 0.3% of the consultations patients were tested on extragenital sites.

**Conclusion:**

Almost 20% of the patients returned for a CT TOC, especially at GP practices in low SES areas. For NG, 1 out of 4 patients returned for a TOC. Retesting rates were low for both CT (24%) and NG (15%), (re)infections including extragenital infections may be missed. Efforts are required to focus TOC and increase retesting practices of GPs in order to improve CT/NG control.

## Introduction

*Chlamydia trachomatis* (CT) and *Neisseria gonorrhoeae* (NG) are the most prevalent bacterial sexually transmitted infections (STIs) diagnosed worldwide [1]. General practitioners (GPs) have a major role in STI healthcare, since most STIs are diagnosed by GPs and STI clinics [2–6].

A test of cure (TOC) within 3–5 weeks after the completion of CT treatment is internationally not recommended because of possible false-positive results leading to overtreatment [7–9]. The British Association for Sexual Health and HIV in cooperation with the Royal College of General Practitioners, as well as the 2012 European guideline on the diagnosis and treatment of gonorrhoea in adults, recommend a TOC for all patients who tested positive for NG two weeks after treatment due to the increasing antimicrobial resistance of NG [10, 11]. However, the American Centers for Disease Control and Prevention only recommend an NG TOC in the case of oropharyngeal NG when treated with an alternative regimen [9]. The Dutch general practitioner guideline does not recommend a CT TOC nor an NG TOC, except for specifically indicated cases [12].

Repeat infections within 3–12 months after CT/NG diagnoses are common, totaling up to 32% for CT and up to 40% for NG [13, 14]. For effective CT/NG control, retesting CT/NG positives within 3–12 months is recommended by the Centers for Disease Control and Prevention as well as the British Association for Sexual Health and HIV [9, 10]. Moreover, a modeling study suggested that the most effective control strategy for the treatment of resistant NG is to retest NG positives, rather than testing and treating more patients [15]. Furthermore, the Dutch GP guideline has been revised since 2013 and recommends that GPs advise CT-positive patients to consider retesting within a year [12]. However, no recommendations are given for retesting NG-positive patients [12].

The majority of CT and NG positives seen by GPs are not retested [16–18]. However, retesting rates may have been underestimated, as patients could have a TOC or a retest at another STI care provider like the STI clinic or the hospital [16]. Insight into TOC and retesting rates, including associated factors among GPs and patients, is vital to inform and improve CT and NG control.

Extragenital CT infections are common among men who have sex with men (MSM) and women [19]. International guidelines recommend extragenital testing based on indication; that is, self-report of anal sex and symptoms [7, 10]. However, studies showed that extragenital CT infections are common among MSM and women without indication, which suggests routine screening at extragenital sites among MSM and women [19, 20]. According to the Dutch GP guideline, additional anorectal CT/NG testing in MSM and an indication of anal sex or anal symptoms is recommended [12]. For NG, additional oropharyngeal testing should be performed for commercial sex workers and MSM who report oral sex or oropharyngeal symptoms. According to recent studies among GPs in the Netherlands, extragenital CT and NG testing was rarely performed [2, 18]. However, an earlier Dutch study estimated that ~9% of male patients with a STI-related GP visit are MSM, for whom extragenital testing is recommended [12, 21].

The socioeconomic status (SES) of patients has been linked to various infectious diseases like CT and NG [2, 18, 22]. However, it remains unknown whether TOC and retesting practices differ between GP practices in low, middle and high SES areas. Such analyses that include the SES of GP practice areas could be helpful in advising GPs about their TOC and retest practices to optimize CT and NG control at a local level.

Here, we assessed CT and NG TOC and retesting practices of GP practices in different SES areas, taking account of TOC and retesting of patients at other STI care providers as well as extragenital testing, in order to inform and optimize local test practices of GP practices.

## Methods and materials

### Ethics statement

The data were obtained from medical records in a fully anonymized and de-identified manner and none of the researchers had access to patient identifying information. The study protocol was exempt from formal medical-ethical approval under prevailing laws in the Netherlands as it concerns an retrospective observational study using anonymous data only (as stated by the National Central Committee for Human Studies: www.ccmo.nl and in the conduct of good behavior in research www.federa.org).

### Study population

**Fig 1:**
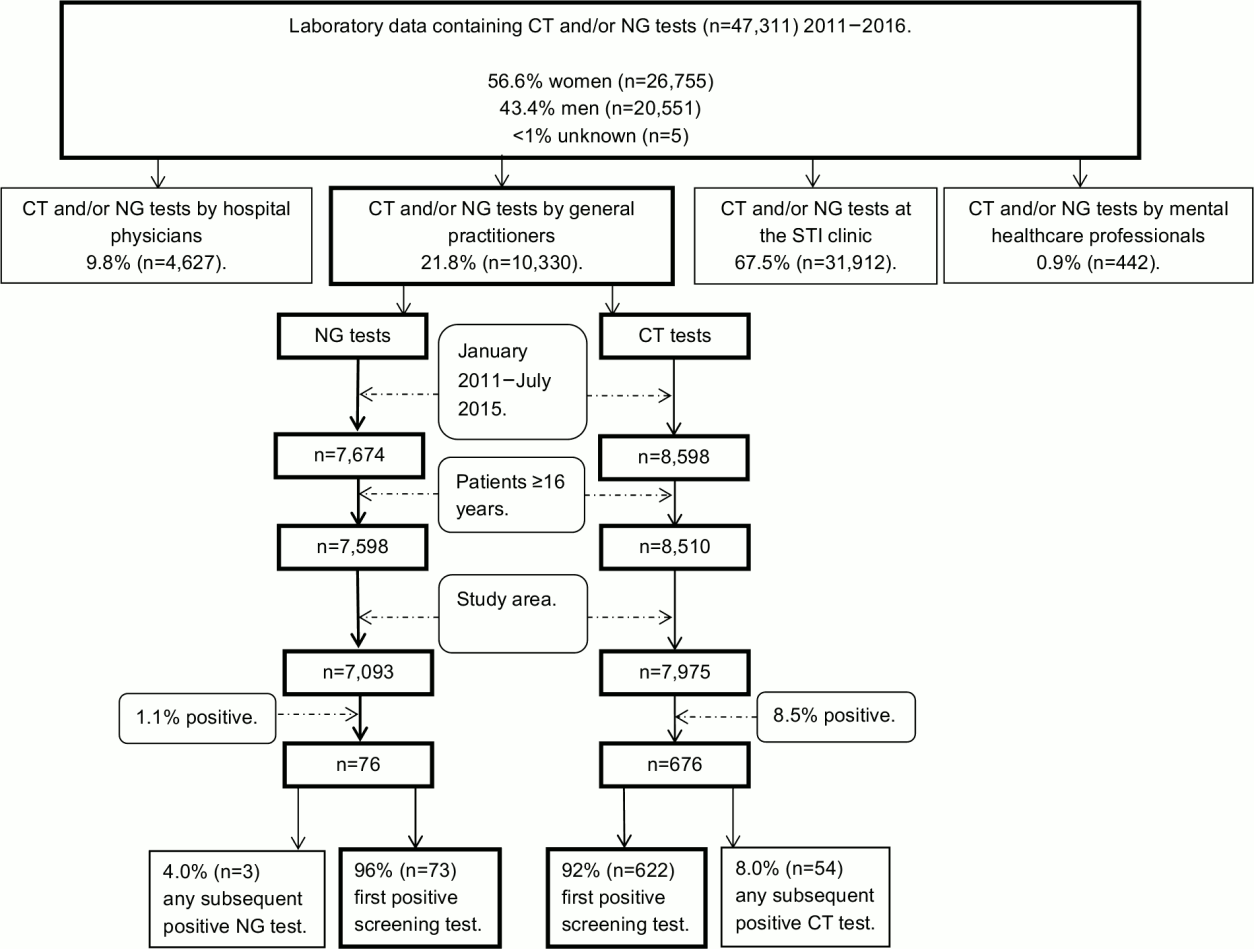
Flowchart of *Chlamydia trachomatis*- and *Neisseria gonorrhoeae*-positive screening tests between January 2011 and July 2015. CT, *Chlamydia trachomatis*; NG, *Neisseria gonorrhoeae*; STI, sexually transmitted infection.

All CT and/or NG laboratory tests of patients ≥16 years were obtained from the database of the regional medical microbiology laboratory of Maastricht University Medical Center (January 2011–July 2016; n = 47,311). The dataset consisted of tests from all four STI care providers, as shown in Fig 1. The study area comprised municipalities surrounding the regional laboratory (Maastricht, Eijsden-Margraten and Valkenburg aan de Geul).

For the purpose of this study, a GP practice was defined as one or more GPs sharing the same postal code and performing CT and/or NG tests. The study area consisted of 48 GP practices. We estimated whether GP practices in the study area sent their CT and NG tests to the regional laboratory. In case of low numbers of CT tests (≤40) per GP practice, or a notable downward trend between 2011 and 2016, GP practices were contacted by telephone to confirm whether they indeed sent their test requests to the regional laboratory. We contacted 20 GP practices in our study area, of which 9 GP practices reported that they sent their tests to another laboratory. As a consequence, the data set covered 81% (39/48) of the GP practices in our study area, ensuring acceptable laboratory coverage. All 48 GP practices were included in the analyses.

Dutch SES scores based on income, education level and employment were extracted from the Netherlands Institute for Social Research (http://www.scp.nl) per four-digit postal code area. The SES score of the GP practice area was determined on the basis of the four-digit postal code of the GP practice. The SES score of the patients’ residential area was based on the four-digit postal code of the patient.

### Definitions

The first positive CT or NG test of a patient was defined as the positive screening test. For those patients identified as having a positive screening test, we assessed five outcome measures: (1) a ‘TOC’ was defined as the first test within three months of the positive screening test carried out by the same GP practice (the period of three months was based on international guidelines advising a retest three months after CT diagnosis [9, 10]); (2) ‘TOC at another STI care provider’ was defined as the first test within three months of the positive screening test carried by other STI care providers; that is, other GP practices, STI clinics or hospital physicians; (3) ‘retesting’ was defined as the first test within 3–12 months after the positive screening test carried out by the same GP practice; (4) ‘retesting at another STI care provider’ was defined as the first test within 3–12 months of the positive screening test carried out by other STI care providers; that is, other GP practices, STI clinics or hospital physicians and (5) ‘extragenital testing’ was defined as the proportion of consultations in which anorectal and oropharyngeal tests were performed by GPs in the period 2011–2015.

TOC and retesting rates were calculated per positive screening test. We excluded CT-positive (n = 138) and NG-positive (n = 20) screening tests and any other tests of patients that were performed during the last year of the data collection (July 2015–July 2016) to ensure the same window of opportunity for retesting among all patients. Positive scores for TOC, retesting and extragenital testing were calculated by the proportion that tested positive at the first TOC or retest, or at extragenital locations.

### Study cohort and statistical analysis

In the period of January 2011 to July 2015, there were 8,014 GP testing consultations. In 88% (n = 7,054) of the consultations, patients were tested for both CT and NG; 11.5% (n = 921) were tested for CT only and 0.5% (n = 39) for NG only.

Analyses were stratified for positive CT and NG screening tests. This procedure resulted in a baseline cohort of 622 CT-positive and 73 NG-positive screening tests (Fig 1), in which every positive screening test in the analysis dataset represents one patient.

To assess factors associated with (1) TOC and (2) retesting, univariate and multivariate logistic regression analyses were performed. The factors assessed were sex, age (<25, ≥25), screening test result (tested for CT only, tested positive for CT and negative for NG, tested positive for CT and NG), TOC (yes, no) for the outcome retest only, SES of patients’ residential area (low, medium, high SES, bases on tertiles), calendar year of screening (<2013, ≥2013; the Dutch GP guideline was revised in 2013), number of tests on a GP practice level (continuous with an increment of 10 tests), urbanization of GP practice area (urban, non-urban), SES of GP practice area (low, medium, high SES, based on tertiles), distance between GP practice and STI clinic (<3 km, ≥3 km), distance between GP practice and laboratory (<3 km, ≥3 km), distance between GP practice and patient (<3 km, ≥3 km), and number of employed GPs per GP practice (1, >1).

Based on existing literature [2, 16–18, 23], multivariate analyses were adjusted for the following factors: sex, age and number of tests per GP practice. The SES of the GP practice area was also included in the multivariate model, as this factor was our main interest.

For all analyses, factors with p<0.10 in the univariate model were included through the backward stepwise method in the multivariate model. Odds ratios and 95% confidence intervals (95% CI) were calculated for the univariate and multivariate models. Analyses were performed using SPSS V21 (IBM SPSS Statistics for Windows, IBM Corporation, Armonk, New York, USA). A p value of <0.05 was considered statistically significant.

## Results

### Chlamydia trachomatis

#### TOC

**Fig 2:**
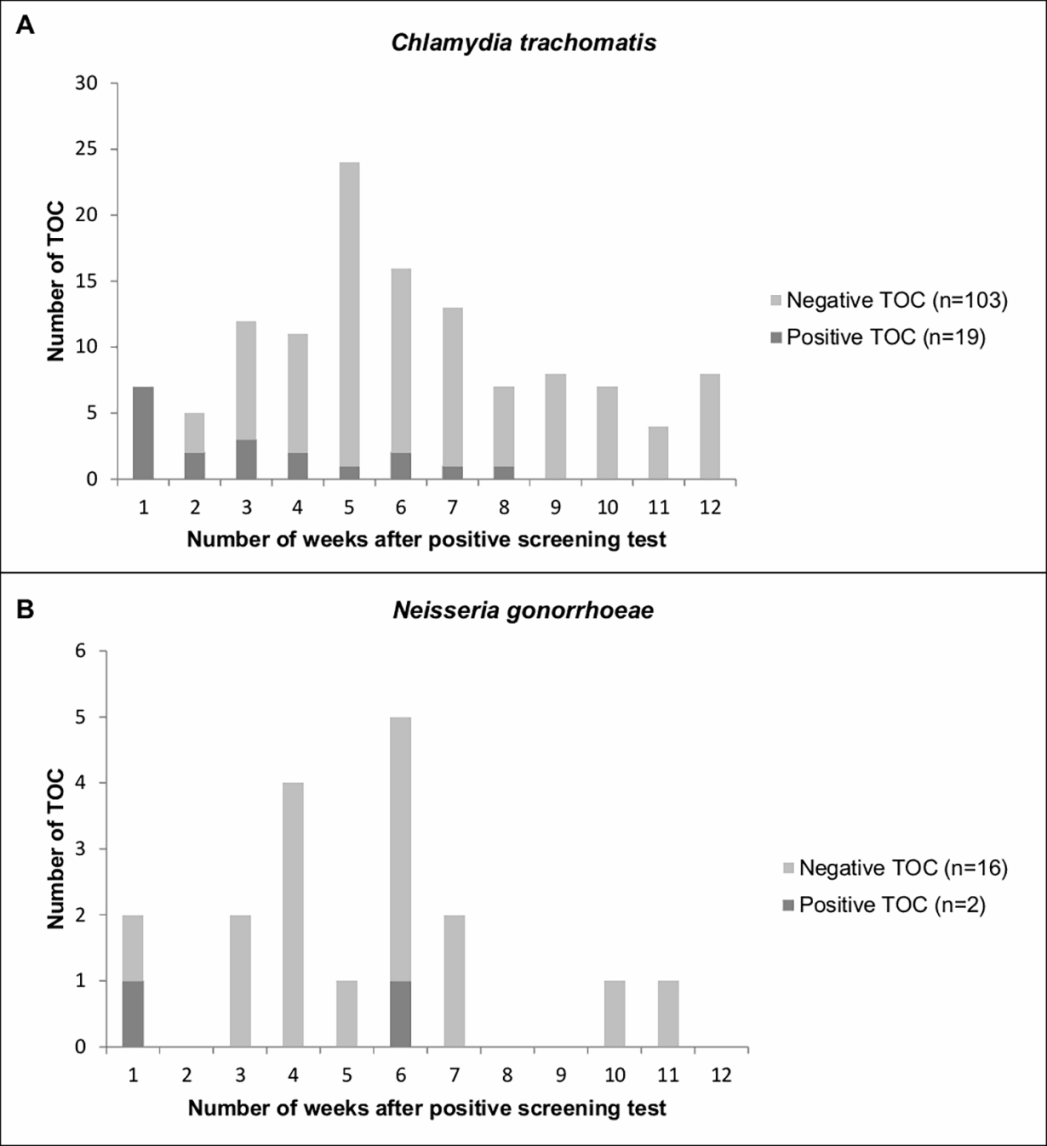
Distribution of the number of tests of cure and positivity per week. (A) The number of tests of cure per week for *Chlamydia trachomatis* based on the positive screening test (n = 622). Two tests of cure were performed in week 13, for clarity reasons these were added to week 12. (B) The number of tests of cure per week for *Neisseria gonorrhoeae* based on the positive screening test (n = 73). TOC, test of cure.

Of our baseline cohort (622 CT-positive patients), 19.6% had a TOC within 3 months (122/622). Of this number, 15.6% tested positive (19/122; Fig 2A). Of the patients with a positive TOC (n = 19), 63.2% (12/19) tested positive within 3 weeks, whereas the minimum time for performing a CT TOC is at least 3 weeks [7–9]. The median time to a TOC was 36 days (interquartile range (IQR) 28–54).Factors independently associated with TOC in multivariate analyses were patients having a younger age (<25 year) and GP practice areas with low and medium SES, respectively (Table 1).

**Table 1:** Factors associated with test of cure and retest results among general practices in the Netherlands, based on the first positive *Chlamydia trachomatis* screening test between January 2011 and July 2015.

	Patients with positive screening test	TOC (<3 months)			Retest (3–12 months)		
	% (n)	% (n)	OR (95% CI)	Adj. OR (95% CI)^a^	% (n)	OR (95% CI)	Adj. OR (95% CI)^a^
Overall	100 (622)	19.6 (122)			23.8 (148)		
**Sex**							
Men	39.2 (244)	16.0 (39)	1	1	22.1 (54)	1	1
Women	60.8 (378)	22.0 (83)	1.48 (0.97–2.25)	1.37 (0.89–2.11)	24.9 (94)	1.17 (0.80–1.71)	1.13 (0.76–1.69)
**Age**							
<25	50.3 (313)	23.0 (72)	**1.55 (1.04–2.31)**	**1.56 (1.03–2.35)**	24.0 (75)	1.03 (0.71–1.49)	0.98 (0.67–1.44)
≥25	49.7 (309)	16.2 (50)	1	1	23.5 (73)	1	1
**Screening test result**							
Only CT+	8.4 (52)	15.4 (8)	1	ns	5.8 (3)	1	1
CT+ and NG-	86.5 (538)	19.7 (106)	1.35 (0.62–2.95)	ns	25.1 (135)	**5.47 (1.68–17.84)**	**5.70 (1.74–18.71)**
CT+ and NG+	5.1 (32)	25.0 (8)	1.83 (0.61–5.50)	ns	31.3 (10)	**7.42 (1.86–29.65)**	**7.20 (1.78–29.04)**
**TOC**							
No	80.4 (500)	na	na	na	21.8 (109)	1	1
Yes	19.6 (122)	na	na	na	32.0 (39)	**1.69 (1.09–2.61)**	**1.58 (1.01–2.47)**
**SES of patients’ residential area**							
Low	31.6 (191)	20.4 (39)	1.53 (0.90–2.59)	ns	24.1 (46)	1.02 (0.64–1.62)	ns
Medium	34.9 (211)	22.3 (47)	**1.71 (1.03–2.85)**	ns	23.2 (49)	0.97 (0.62–1.53)	ns
High	33.4 (202)	14.4 (29)	1	ns	23.8 (48)	1	ns
**Calendar year of screening**							
<2013	37.8 (235)	19.6 (46)	1	ns	25.5 (60)	1	ns
≥2013	62.2 (387)	19.6 (76)	1.00 (0.67–1.51)	ns	22.7 (88)	0.86 (0.59–1.25)	ns
**Number of CT tests per GP practice**							
Continuous (increment of 10 tests)	na	na	**1.19 (1.02–1.38)**	1.13 (0.95–1.33)		1.11 (0.96–1.28)	1.12 (0.96–1.31)
**Urbanization of GP practice area**							
Urban	84.9 (528)	19.3 (102)	1	ns	23.9 (126)	1	ns
Non-urban	15.1 (94)	21.3 (20)	1.13 (0.66–1.94)	ns	23.4 (22)	0.98 (0.58–1.64)	ns
**SES of GP practice area**							
Low	28.6 (178)	23.6 (42)	**1.90 (1.16–3.13)**	**1.83 (1.08–3.10)**	21.9 (39)	1.03 (0.64–1.64)	ns
Medium	31.7 (197)	22.8 (45)	**1.82 (1.12–2.97)**	**1.89 (1.15–3.10)**	28.4 (56)	1.45 (0.94–2.24)	ns
High	39.7 (247)	14.2 (35)	1	1	21.5 (53)	1	ns
**Distance** **GP practice–STI clinic**							
<3 km	39.9 (248)	19.0 (47)	0.93 (0.62–1.40)	ns	23.4 (58)	0.96 (0.66–1.41)	ns
≥ 3 km	60.1 (374)	20.1 (75)	1	ns	24.1 (90)	1	ns
**Distance** **GP practice–laboratory**							
<3 km	20.4 (127)	18.9 (24)	0.94 (0.58–1.55)	ns	28.3 (36)	1.35 (0.87–2.10)	ns
≥ 3 km	79.6 (495)	19.8 (98)	1	ns	22.6 (112)	1	ns
**Distance** **GP practice–patient**							
<3 km	63.5 (395)	20.5 (81)	1.17 (0.77–1.78)	ns	24.1 (95)	1.04 (0.71–1.53)	ns
≥ 3 km	36.5 (227)	18.1 (41)	1	ns	23.3 (53)	1	ns
**Number of employed GPs**							
1	13.3 (83)	16.9 (14)	0.81 (0.44–1.49)	ns	13.3 (11)	1	ns
>1	86.7 (539)	20.0 (108)	1	ns	25.4 (137)	**2.23 (1.15–4.33)**	ns

Adj., adjusted; OR, odds ratio; TOC, test of cure; CT, *Chlamydia trachomatis*; NG, *Neisseria gonorrhoeae*; SES, socioeconomic status; GP, general practitioner; na, not applicable; ns, not significant.

^a^ Adjusted for sex, age and number of Chlamydia trachomatis tests per GP practice.

#### TOC at another STI care provider

**Fig 3:**
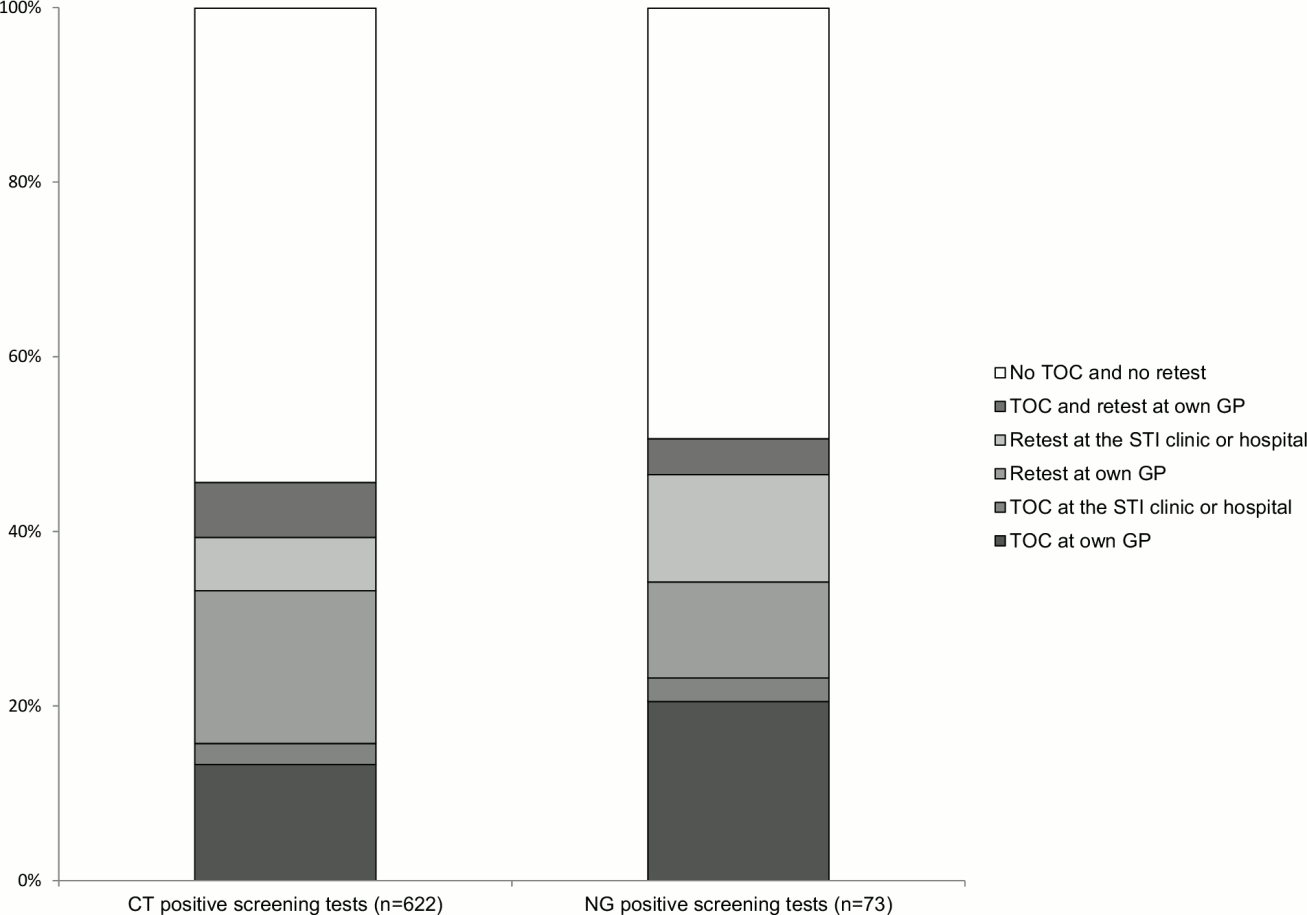
Distribution of *Chlamydia trachomatis* and *Neisseria gonorrhoeae* test of cure and retest results among patients with a positive screening test at the general practice. CT, *Chlamydia trachomatis*; NG, *Neisseria gonorrhoeae*; TOC, test of cure; GP, general practice; STI, sexually transmitted infection.

Of our baseline cohort, 2.4% (15/622) had a TOC at another STI care provider (none at another GP practice, 11 at the STI clinic and 4 at the hospital). Of this number, 33.3% (5/15) tested positive (Fig 3).

#### Retesting

A retest within 3–12 months was performed in 23.8% (148/622) of the CT patients. Of this number, 12.2% tested positive (18/148). The median time to a retest was 182 days (IQR 125–265). Factors independently associated with retesting in multivariate analyses were patients who screened positive for CT and negative for NG, patients who screened positive for both CT and NG, and patients who had a TOC (Table 1).

#### Retesting at another care provider

Retesting at other STI care providers comprised 6.1% (38/622) (none at another GP practice, 32 at the STI clinic and 6 at the hospital). Of this number, 15.8% (6/38) tested positive (Fig 3).

#### Extragenital testing

Testing at extragenital sites was performed in 0.3% of the CT consultations (25/7975). Of this number, 7 were on anorectal sites (positivity 14.3%; 1/7) and 18 were on oropharyngeal sites (positivity 0%; 0/18).

### Neisseria gonorrhoeae

#### TOC

Of our baseline cohort of 73 patients, 24.7% had a TOC within 3 months (18/73). Of this number, 11.1% (2/18) tested positive. The median time to a TOC was 33 days (IQR 21–42). One patient had a positive TOC after 5 days of the positive screening test and one patient had a positive TOC after 41 days of the positive screening test (Fig 2B). In multivariate analyses, no factors were independently associated with TOC (Table 2).

**Table 2:** Factors associated with test of cure and retest results among general practices in the Netherlands, based on the first positive *Neisseria gonorrhoeae* screening test between January 2011 and July 2015.

	Patients with positive screening test	TOC (<3 months)			Retest (3–12 months)		
	% (n)	% (n)	OR (95% CI)	Adj. OR (95% CI)^a^	% (n)	OR (95% CI)	Adj. OR (95% CI)^a^
Overall	100 (73)	24.7 (18)			15.1 (11)		
**Sex**							
Men	52.1 (38)	13.2 (5)	1	1	13.2 (5)	1	1
Women	47.9 (35)	37.1 (13)	**3.90 (1.22–12.49)**	3.46 (0.98–12.23)	17.1 (6)	1.37 (0.38–4.95)	0.60 (0.12–3.11)
**Age**							
<25	42.5 (31)	38.7 (12)	**3.79 (1.23–11.69)**	3.19 (0.92–11.00)	19.4 (6)	1.78 (0.49–6.46)	0.54 (0.10–3.00)
≥25	57.5 (42)	14.3 (6)	1	1	11.9 (5)	1	1
**Screening test result**^**b**^							
NG+ and CT-	56.2 (41)	19.5 (8)	1	ns	7.3 (3)	1	1
NG+ and CT+	43.8 (32)	31.3 (10)	1.88 (0.64–5.49)	ns	25.0 (8)	**4.22 (1.02–17.50)**	**9.77 (1.40–68.12)**
**TOC**							
No	75.3 (55)	na	na	na	14.5 (8)	(1 ref)	ns
Yes	24.7 (18)	na	na	na	16.7 (3)	1.18 (0.28–5.00)	ns
**SES of patients’ residential area**							
Low	20.5 (15)	26.7 (4)	1.27 (0.30–5.48)	ns	6.7 (1)	0.41 (0.04–4.06)	ns
Medium	42.5 (31)	25.8 (8)	1.22 (0.36–4.09)	ns	19.4 (6)	1.38 (0.35–5.52)	ns
High	37.0 (27)	22.2 (6)	1	ns	14.8 (4)	1	ns
**Calendar year of screening**							
<2013	39.7 (29)	34.5 (10)	1	ns	20.7 (6)	1	ns
≥2013	60.3 (44)	18.2 (8)	0.42 (0.14–1.25)	ns	11.4 (5)	0.49 (0.14–1.79)	ns
**Number of NG tests per GP practice**							
Continuous (increment of 10 tests)	na	na	3.34 (0.11–97.77)	2.51 (0.04–148.14)		1.81 (0.03–103.83)	0.91 (0.00–232.77)
**Urbanization of GP practice area**							
Urban	87.7 (64)	25.0 (16)	1	ns	14.1 (9)	1	ns
Non-urban	12.3 (9)	22.2 (2)	0.86 (0.16–4.55)	ns	22.2 (2)	1.75 (0.31–9.77)	ns
**SES of GP practice area**							
Low	32.9 (24)	20.8 (5)	0.90 (0.22–3.63)	1.32 (0.29–6.07)	8.3 (2)	0.58 (0.09–3.82)	ns
Medium	37.0 (27)	29.6 (8)	1.43 (0.39–5.23)	1.81 (0.39–8.30)	22.2 (6)	1.81 (0.40–8.26)	ns
High	30.1 (22)	22.7 (5)	1	ns	13.6 (3)	1	ns
**Distance** **GP practice–STI clinic**							
<3 km	46.6 (34)	26.5 (9)	1.20 (0.41–3.48)	ns	8.8 (3)	0.38 (0.09–1.55)	ns
≥3 km	53.4 (39)	23.1 (9)	1	ns	20.5 (8)	1	ns
**Distance** **GP practice–laboratory**							
<3 km	20.5 (15)	33.3 (5)	1.73 (0.50–5.97)	ns	40.0 (6)	**7.07 (1.78–28.12)**	**14.81 (1.91–114.82)**
≥3 km	79.5 (58)	22.4 (13)	1	ns	8.6 (5)	1	1
**Distance** **GP practice–patient**							
<3 km	63.0 (46)	23.9 (11)	0.90 (0.30–2.69)	ns	19.6 (9)	3.04 (0.61–15.27)	ns
≥3 km	37.0 (27)	25.9 (7)	1	ns	7.4 (2)	1	ns
**Number of employed GPs**							
1	19.2 (14)	14.3 (2)	1	ns	7.1 (1)	1	ns
>1	80.8 (59)	27.1 (16)	2.23 (0.45–11.09)	ns	16.9 (10)	2.65 (0.31–22.65)	ns

Adj., adjusted; OR, odds ratio; TOC, test of cure; CT, *Chlamydia trachomatis*; NG, *Neisseria gonorrhoeae*; SES, socioeconomic status; GP, general practitioner; na, not applicable; ns, not significant.

^a^ Adjusted for sex, age and number of Neisseria gonorrhoeae tests per GP practice.

^b^ This factor was aggregated, since all NG patients were also tested for Chlamydia trachomatis.

#### TOC at another STI care provider

Of the 73 patients with a positive NG screening test, 2.7% (2/73) had a TOC at another STI care provider (two at the STI clinic). Of this number, one tested positive (Fig 3).

#### Retesting

A retest within 3–12 months was performed in 15.1% of the NG patients (11/73). Of this number, 0% (0/11) tested positive. The median time to a retest was 224 days (IQR 140–254). Independent factors associated with retesting in multivariate analyses were patients screening positive for both NG and CT, and a distance of <3 km between GP practice and laboratory (Table 2).

#### Retesting at another STI care provider

In 12.3% (9/73) of the patients a retest was performed at another STI care provider (none at another GP practice, seven at the STI clinic and two at the hospital). Of this number, none tested positive (Fig 3).

#### Extragenital testing

Testing at extragenital sites was performed in 0.3% (23/7,093) of the NG consultations. Of this number, 6 were performed on anorectal sites (positivity 0.0%; 0/6) and 17 on oropharyngeal sites (positivity 5.9%; 1/17).

## Discussion

This retrospective cohort analysis of 48 Dutch GP practices shows that a TOC was performed in approximately 20% of the CT patients, especially at GP practices in low SES areas and in patients with a younger age (<25). Furthermore, 1 out of 4 patients had an NG TOC, which is generally not recommended in the Dutch GP guideline. However, an NG TOC is recommended in international guidelines due to increasing antimicrobial resistance of NG [9–11]. Most CT (76%) and NG (85%) patients did not have a retest at their GP. Overall, 12% retested CT positive and 0% retested NG positive. Due to the low number of CT and NG retests, repeat infections were likely missed. A comparable proportion of the NG patients were retested at the GP practice (15%) and at other STI care providers (12%), while the percentages for CT retests were 24% at the GP practice and 6% at other STI care providers. Furthermore, extragenital CT and NG testing was rarely performed at GP practices (0.3%), in which extragenital infections were likely missed.

Several studies have been performed assessing CT/NG test practices among different STI care providers [16, 23–26]. However, these studies only include patient-related factors and lack GP characteristics. We included these factors to draw up recommendations specifically for GPs in order to enhance CT/NG control. Moreover, we provided evidence that the retesting rates of patients initially seen by their GP are underestimated; that is, we showed that the rate of retesting NG patients at other STI care providers (12%) is comparable to the rate of retesting at the GP practice (15%). Despite the small numbers, analyses of NG test practices are necessary, as following up on NG positives seems to be the most effective control strategy for the treatment of resistant NG [15]. Furthermore, we analyzed recent data from before and after the revision to the Dutch GP guideline in order to assess current retesting practices of GPs. However, no difference between the two time periods was observed (Tables 1 and 2). Finally, a further strength is that we assessed whether GP practices in our study area indeed send their tests to the regional laboratory, in which we estimated that 81% (39/48) of the GP practices did.

A general limitation of the study was that information on patients’ sexual behavior and on the characteristics of or reasons for testing was unavailable. Such reasons may include financial reasons. STI tests at the GP are within patients’ deductibles in healthcare insurances, whereas STI tests at the STI clinic are free of charge for risk groups (age <25, MSM and commercial sex workers) [23, 27]. An earlier study in the southeastern part of Limburg showed that CT retesting rates were lower for GPs (23.0%) in comparison with the STI clinic (33.4%) and gynecologists (30.3%) [17]. In addition, we operationalized the outcomes according to international guidelines with a cut-off point of three months. As a result, we were unable to assess whether a CT/NG TOC or retest was also defined as such by the treating GP. A limitation for the outcome TOC was that we were unable to assess whether a CT or NG TOC was justified according to the Dutch GP guideline; that is, in the case of pregnancy, persistent symptoms, reexposure to untreated source or lack of treatment with first choice of treatment [12]. In addition, the use of the same time period for assessing TOC for both CT and NG is debatable. While a TOC for CT is not advisable, a TOC two weeks after NG treatment is internationally recommended [10–14]. However, to enable a comparison between CT and NG, we applied equal TOC periods [12]. Still, the exact time for performing an NG TOC is under debate [28, 29].

We showed that almost 1 in every 5 patients had a CT TOC. Moreover, almost two-thirds of the positive CT TOC were diagnosed within 3 weeks of the positive screening test, which is strongly not recommended and can be false positive results leading to overtreatment [8, 9, 30]. If indicated, a CT TOC should be performed at least 3 weeks post treatment [7, 9, 12]. An Australian study showed that 25% of the patients who tested positive for CT at the GP made a new test request within six weeks, which is also not recommended in the Australian GP guidelines [16]. An NG TOC was performed in almost 1 in 4 patients, which is not recommended in the Dutch GP guideline. However, forgoing a TOC after NG treatment is debatable, as international guidelines recommend a TOC two weeks post treatment for all NG patients [10, 11] or patients with oropharyngeal NG and treated with alternative medication [9]. One patient had a positive TOC after 40 days of the positive screening test, which could be related to treatment failure [11]. Still, no cases of antimicrobial resistance have yet been reported in the Netherlands [15, 31].

A high proportion of CT repeat infections were diagnosed. Other studies showed higher retesting rates among GPs than our study, still retesting rates were low and likely infections were missed [16, 32]. Moreover, due to low rate of extragenital testing also extragenital infections were likely missed, since extragenital infections are common in MSM as well as in women without symptoms in STI clinic settings [19, 21]. However, this remains unclear for the GP population. Similar findings of low extragenital testing among GPs in another Dutch region confirm the likely generalizability of our study [2, 18].

According to the laboratory data (Fig 1), most CT and NG tests were performed by the STI clinic followed by GP practices. In the Netherlands, TOC rates are higher at GP practices compared to STI clinics, whereas retesting rates are higher at STI clinics compared to GP practices [17, 18, 23]. However, in other countries retesting rates seem higher at GP practices than STI clinics [16, 32].

According to previous studies, the number of positive CT and NG tests is highest among patients living in low SES areas [2, 18]. The SES of patients’ living areas was not associated with CT or NG TOC in the current study. However, most patients lived near the GP practice (<3 km; Tables 1 and 2). This fact could explain the higher CT TOC rate of GP practices in low SES areas in our study. Our results show that patients who tested for both CT and NG were more likely to retest for CT and NG in comparison with patients who only tested for CT (Table 1). Patients without an NG test are likely to be a low-risk group or to lack symptoms, as the Dutch GP guideline recommends an additional NG test in high-risk groups or when having symptoms [12]. An NG retest is not mentioned in the Dutch GP guideline [12]. Positivity of NG retesting was 0% in the current study, which may be explained by the low rate of retesting (~15%). Recently, another study among GPs in the Netherlands showed a retest positivity rate for NG of ~23% [18]. As studies which include positivity in NG retesting of GP patients are scarce, more research is needed for NG control optimization [18].

A CT TOC should never be performed within three weeks after CT treatment due to false-positive results what could lead to overtreatment [7–9]. A CT TOC is in general not needed but should be performed (1) in the case of pregnancy, (2) when having persistent symptoms or (3) when there is lack of treatment with first choice treatment, as described in the Dutch GP guideline [12]. An NG TOC is internationally recommended at least two weeks post treatment due to increasing antimicrobial resistance of NG [10, 11]. Unfortunately this is not recommended in the Dutch GP guideline [12]. All CT and NG patients should be retested within three to twelve months, despite that the minimum time of three months is not mentioned in the Dutch GP guideline [12]. Extragenital testing should be performed in all MSM and patients reporting anorectal intercourse or symptoms, as described in the Dutch GP guideline [12].

To facilitate retesting at the GP practice, GPs could consider using modern testing and communication strategies such as e-health. For example, GPs could, in collaboration with their diagnostic laboratory and public health services, send home sampling kits 3 to 12 months post treatment to improve retesting rates. Also automatic small text messages could be used. These two methods have shown to increase retesting rates in specific settings, but not in GP settings yet [23, 33]. Moreover, education and awareness of the importance of CT and NG testing could improve (re)testing rates at the GP practice [34].

## Conclusion

Almost 1 in every 5 CT positives returned for a TOC. Especially GP practices in lower SES areas performed a CT TOC, which is not recommended. Most patients did not have a CT retest, although the high CT positivity at retesting (12%) demonstrates the need to encourage retesting of CT positives. The proportion of retesting for NG positives was also low (15%). Moreover, only 0.3% of the CT/NG consultations had patients tested on extragenital sites. As a result, infections or reinfections could have been missed.

A TOC for CT is not needed, whereas a TOC for NG should be performed. All CT and NG patients should be retested within three to twelve months. GPs should perform extra genital testing in all MSM and in all patients with anorectal intercourse or symptoms. The Dutch GP guideline needs to be reconsidered, especially regarding NG TOC and retesting as it is inconsistent with international guidelines.
